# Shanling Jiangzhi Tea treats hyperuricemia by inhibition of COMT/MAOA signaling pathway and p53/SERPINE1/NLRP3 signaling pathway

**DOI:** 10.3389/fendo.2025.1623111

**Published:** 2025-07-11

**Authors:** Hao-nan Chen, He-zhen Wu, Yan-fang Yang

**Affiliations:** ^1^ Hubei Key Laboratory of Chinese Medicine Resources and Chinese Medicine Chemistry, School of Pharmacy, Hubei University of Traditional Chinese Medicine, Wuhan, Hubei, China; ^2^ Hubei Engineering Research Center of Modern Chinese and Ethnic Medicine, Wuhan, Hubei, China; ^3^ Hubei Shizhen Laboratory, Wuhan, Hubei, China

**Keywords:** Shanling Jiangzhi Tea, hyperuricemia, transcriptomics, network pharmacology, mechanism

## Abstract

**Objective:**

To evaluate the therapeutic efficacy of Shanling Jiangzhi Tea (SLJZ) on hyperuricemia (HUA) mice and to investigate its mechanism.

**Methods:**

A HUA mouse model was established using a combination of uric acid (UA)and potassium oxonate. Following SLJZ intervention, changes in body weight were monitored. Renal lesions and renal fibrosis were assessed via H&E staining and Masson trichrome staining. Serum levels of UA, creatinine (Cr), blood urea nitrogen (BUN), and xanthine oxidase (XOD) were measured to evaluate the UA-lowering effects of SLJZ. Ultra-performance liquid chromatography-quadrupole-time-of-flight mass spectrometry (UPLC-Q-TOF-MS) was employed to identify the bioactive components of SLJZ that entered the bloodstream. Network pharmacology, molecular docking, and transcriptomics analyses were conducted to elucidate the key targets and signaling pathways involved in SLJZ’s therapeutic effects on HUA. Protein expression levels were further validated using immunohistochemistry.

**Results:**

SLJZ significantly reduces the levels of UA, Cr, BUN, and XOD in the blood of HUA mice, alleviates inflammatory cell infiltration, attenuates renal interstitial fibrosis, and demonstrates therapeutic potential for hyperuricemia. RNA-seq analysis reveals that SLJZ reverses 280 HUA-induced differentially expressed genes (DEGs) in the kidneys. Based on the findings from network pharmacology and molecular docking analyses, SERPINE1, p53, NLRP3, COMT, and MAOA are identified as potential key proteins involved in SLJZ’s treatment of HUA. Kit-based assays indicate that SLJZ increases dopamine (DA) levels in the kidneys of mice while reducing the levels of interleukin-6 (IL-6), interleukin-1β (IL-1β), and tumor necrosis factor-α (TNF-α). Immunohistochemical results confirm that SERPINE1, p53, NLRP3, COMT, and MAOA are significantly upregulated in the kidneys of HUA model mice, but their expression is normalized following SLJZ intervention.

**Conclusion:**

SLJZ exhibits a significant anti-HUA effect by inhibiting the COMT/MAOA signaling pathway and the p53/SERPINE1/NLRP3 signaling pathway. Through these mechanisms, SLJZ is involved in the DA metabolic process, modulates the inflammatory response mediated by the SERPINE1 fibrinolytic system, alleviates renal tubulointerstitial fibrosis, and mitigates oxidative stress, thereby exerting therapeutic effects on HUA.

## 1 Introduction

Hyperuricemia (HUA) is a chronic metabolic disorder resulting from abnormal purine metabolism, characterized by excessive production of uric acid (UA) or impaired UA excretion. It has become the fourth most common metabolic disease after hypertension, hyperlipidemia and hyperglycemia (1). Epidemiological investigations have demonstrated that changes in lifestyle and dietary patterns are associated with a global increase in the incidence of HUA (2). UA is a product of purine metabolism, mainly excreted through the kidneys and intestines following the breakdown of purines from both cellular metabolism and dietary intake in the liver. In healthy individuals, UA levels are maintained in equilibrium; however, metabolic disturbances can lead to its accumulation in the body, resulting in HUA (3). When HUA advances to a certain stage, it may induce various complications, including gout and renal impairment. In severe cases, it can cause joint deformities, renal failure, or cardiovascular and cerebrovascular events (4).

Currently, the clinical management of HUA primarily relies on xanthine oxidase inhibitors such as allopurinol (AP) and febuxostat to suppress UA production (5), while Benzbromarone serves as a representative agent for enhancing UA excretion. However, AP is associated with a broad spectrum of adverse effects, including headache, diarrhea, hepatic toxicity, and renal impairment (6). Additionally, febuxostat exhibits a relatively elevated risk of cardiovascular events. Globally, traditional herbal medicines have garnered significant attention for the treatment of HUA and its complications owing to their minimal side effects and favorable therapeutic efficacy. Consequently, the exploration and development of alternative and complementary therapies for HUA, including traditional Chinese medicine, have emerged as a promising research focus.

Shanling Jiangzhi Tea (SLJZ) is formulated with five traditional Chinese medicines: Ganoderma lucidum, Hawthorn, Cassia seed, Mulberry leaf and Mulberry fruit. This compound has been widely used in clinical practice for years and has demonstrated significant therapeutic effects on metabolic diseases. Specifically, Hawthorn aids digestion, resolves blood stasis, and contributes to lipid-lowering and hypotensive effects within the formula. Ganoderma lucidum tonifies qi, calms the mind, and enhances immunity. Mulberry fruit, Mulberry leaf, and Cassia seed nourish the liver and kidneys. These five herbs are commonly employed to address diseases associated with metabolic disturbances or endocrine imbalances. As edible and medicinal materials, they have few documented adverse reactions. Recently, clinical observations have indicated that SLJZ exhibits therapeutic potential for HUA, but the precise mechanism of action remain unclear.

In this study, potassium oxonate and UA were employed as modeling agents to simulate the pathogenesis characterized by increased UA production and decreased excretion. A murine HUA model was established to evaluate the therapeutic efficacy of SLJZ on HUA. Furthermore, we investigated the potential mechanisms of SLJZ by integrating network pharmacology analysis with transcriptome sequencing data, and validated the interactions between active compounds and their potential targets as well as the expression levels of target proteins using molecular docking and immunohistochemistry techniques. This study provides a solid foundation for comprehensively elucidating the pharmacodynamics and underlying mechanisms of SLJZ in treating HUA. Additionally, the research methodology offers a valuable reference for pharmacodynamic studies of other traditional Chinese medicine formulations.

## 2 Materials and methods

### 2.1 Reagents and materials

Potassium oxonate (PO, JS250846) and uric acid (UA, IS236490) were purchased from Shanghai Yuan Ye Biotechnology Co., Ltd. (Shanghai, China). Allopurinol (AP, H31020334) was purchased from Shanghai Xinyi Wanxiang Pharmaceutical Co., Ltd. (Shanghai, China). Kits for the determination of uric acid (UA), creatinine (Cr), and blood urea nitrogen (BUN) were purchased from Nanjing Jiancheng Bioengineering Institute (Nanjing, China). Xanthine oxidase (XOD), dopamine (DA), tumor necrosis factor-α (TNF-α), interleukin-6 (IL-6), and interleukin-1β (IL-1β) detection kits were purchased from Wuhan Elabscience Biotechnology Co., Ltd. (Wuhan, China). H&E staining solution kits and Masson staining solution kits were purchased from Pinofei Biotechnology Co., Ltd. (Wuhan, China). Anti-p53 antibody and anti-MAOA antibody were purchased from Jiangsu Qinke Biological Research Center Co., Ltd. (Liyang, China). Anti-COMT antibody, anti-SERPINE1 antibody, and anti-NLRP3 antibody were purchased from Wuhan Sanying Biotechnology Co., Ltd. (Wuhan, China). Horseradish peroxidase (HRP)-conjugated goat anti-mouse IgG or anti-rabbit IgG was purchased from Wuhan Sanying Biotechnology Co., Ltd. (Wuhan, China).

### 2.2 Animal experiment

SPF ICR male mice (8 weeks old, 20 ± 2 g) were purchased from Henan Skibbes Biotechnology Co., Ltd. (Laboratory Animal Production License No. SCXK (Yu) 2020-0005). The mice were acclimatized for 1 week under controlled conditions of 25 °C, with ad libitum access to standard rodent chow and water throughout the experiment. All experimental procedures adhered to the guidelines for the care and use of laboratory animals and were approved by the Laboratory Animal Ethics Committee of Wuhan Hualianke Biotechnology Co, Ltd. (approval No. HLK-20250427-001).

After the acclimatization feeding period, the mice were randomly divided into 6 groups (n=12/group). The normal control group (Control) received daily intraperitoneal injections of normal saline as a control. The other groups were injected intraperitoneally with potassium oxonate (250 mg/kg/d) and UA (150 mg/kg/d) to establish the HUA mouse model. After 1 h of modeling, the model group (Model) was administered 0.5% CMC-Na solution via gavage as a control, while the remaining groups received interventions for 14 days: low-dose SLJZ (0.832 g/kg/d), medium-dose SLJZ (1.664 g/kg/d), high-dose SLJZ (3.328 g/kg/d), and allopurinol (AP, 10 mg/kg/d). The SLJZ dosages corresponded to 1x, 2x, and 4x the adult human daily dose. Body weight was recorded every 2 days throughout the experiment. 2 h after the final administration, the mice were euthanized, and blood and kidneys were collected for further analysis.

### 2.3 Biochemical parameters

The collected blood samples were stored overnight in a refrigerator at -4°C. On the following day, the samples were centrifuged at 12,000 rpm for 10 min, and the supernatant was carefully collected. The levels of UA, Cr, BUN, and XOD in the supernatant were measured using commercially available kits according to the manufacturer’s instructions.

### 2.4 Renal histopathology

Renal tissue sections were stained with H&E to observe the basic structure, cell morphology and inflammatory damage of kidney tissue, while Masson staining was used to analyze renal fibrosis (7). The collected renal tissues were fixed in 10% paraformaldehyde for 24 h, embedded in paraffin, and sectioned (8 μm) for H&E or Masson staining. The sections were visualized and photographed under a microscope (200×).

### 2.5 Network pharmacology analysis

#### 2.5.1 blood components

From the model group and the high-dose SLJZ group, 200 µL of serum was collected respectively. To fully precipitate proteins, 1 mL of methanol was added, followed by centrifugation. The supernatant was then dried under nitrogen flow at 37°C. Subsequently, the residue was dissolved in 200 µL of methanol, filtered through a 0.22 µm microporous filter membrane, and injected for analysis. Analysis was performed using a Waters Ultra Performance Liquid Chromatograph (Waters Corporation, USA) coupled with a Waters Xevo G2-XS QTOF time-of-flight mass spectrometer (Waters Corporation, USA). Sample separation was achieved on an Ultimate UHPLC XB-C18 column (100 × 2.1 mm, 1.8 µm) with 2 µL injection volume. The mobile phase consisted of water containing 0.1% formic acid (A) and acetonitrile containing 0.1% formic acid (B), delivered at a flow rate of 0.3 mL/min according to the following gradient program: 0–15 min, 95% A; 15–55 min,95%-65%A; 55–65 min,65%-35%A; 65–75 min,35%-10%A; 75–80 min, 10% A. The detection wavelength was set at 254 nm, the column temperature at 30°C, and the ESI ion source was used for scanning under both positive and negative ion modes. The mass-to-charge ratio scanning range was *m/z* 0-1000. The ESI ion source temperature was 100°C, the desolvation gas (N_2_) flow rate was 800 L/h, the desolvation gas temperature was 400°C, the electrospray voltage was 3000 V, the cone voltage was 20 V, the curtain gas pressure was 40 psi, and the auxiliary gas pressure was 50 psi. The low mass collision energy was set at 10–40 V, and the high mass collision energy was set at 40–120 V. Real-time data calibration was performed by continuously injecting leucine enkephalin solution (flow rate: 0.1 mL/min, *m/z* 554.2615) under negative ion mode. The blood components of SLJZ were analyzed using MassLynx V4.1 software.

#### 2.5.2 Target prediction of SLJZ for the treatment of HUA

The SMILES of blood components were retrieved from the PubChem database (https://pubchem.ncbi.nlm.nih.gov/) and subsequently imported into the SwissTargetPrediction database (http://swisstargetprediction.ch/) for predicting their potential protein targets. Targets with a prediction possibility greater than 0 were selected, and all identified targets were summarized and duplicates removed to obtain the final targets of the components. Using “Hyperuricemia” as the keyword, disease-related targets were retrieved from the Genecards (https://www.genecards.org/), DisGeNET database (http://www.disgenet.org/) and NCBI database (https://www.ncbi.nlm.nih.gov/). All disease-related targets were summarized, and duplicates were removed to generate the final list of disease-related targets. The intersection between the disease-related targets and the blood component targets was then determined to identify the potential therapeutic targets of SLJZ in treating HUA.

#### 2.5.3 Protein-protein interactions analysis

The intersection targets were input into the STRING database (https://string-db.org/) for PPI analysis. The confidence score threshold was set to 0.4 to ensure reliable interactions. Network information was exported in “TSV” format and subsequently imported into Cytoscape 3.9.1 software to construct the PPI network diagram.

#### 2.5.4 GO and KEGG analysis

The potential therapeutic targets of SLJZ for HUA were uploaded to the DAVID database (https://davidbioinformatics.nih.gov/) for GO analysis, encompassing biological process (BP), cellular component (CC), molecular function (MF), as well as KEGG pathway enrichment. The species was specified as “Homo sapiens”, and the identifier was set to “Gene Symbol”. Subsequently, the enrichment results were imported into the Microbiomics platform for visualization, and the top 10 GO enrichment items and KEGG pathways with the highest significance (*P* < 0.05) were presented in the form of bubble charts.

### 2.6 Transcriptome analysis of mouse kidney samples

#### 2.6.1 Total RNA extraction, cDNA library preparation and sequencing

RNA-seq analysis was conducted on three mouse kidney samples from the control, model, and SLJZ high-dose groups, respectively. Total RNA was extracted from mouse kidney tissues using Trizol reagent. mRNA was isolated from the extracted total RNA via A-T base pairing with Oligo(dT)-coated magnetic beads. Subsequently, mRNA was fragmented into approximately 300 bp segments by adding fragmentation buffer. Under the action of reverse transcriptase, the mRNA served as a template for first-strand cDNA synthesis using random primers, followed by second-strand synthesis to form a stable double-stranded cDNA structure. After end repair, A-tailing, adapter ligation and size selection, the final library was PCR-amplified using the selected fragments. Finally, transcriptome sequencing was performed on the NonaSeq X Plus platform.

#### 2.6.2 Differential expression genes and pathway enrichment analysis

The expression differences of genes between groups were analyzed and screened to identify DEGs. DESeq2 software was used for differential gene analysis, and the criteria for screening DEGs were set as follows: *P*≤ 0.05 and |log2 FC| ≥ 1. A gene was considered a DEG when both conditions were satisfied. Comparative analyses were conducted on DEGs between the control group and model group, as well as between the SLJZ group and model group, to identify shared genes. These shared genes represent the potential therapeutic targets of SLJZ for treating HUA (8).

#### 2.6.3 GO and KEGG analysis of DEGs

To further elucidate the mechanism of SLJZ in treating HUA, the screened DEGs were imported into the DAVID database for GO analysis and KEGG pathway enrichment. The species was specified as “Homo sapiens”, and the identifier was set to “Gene Symbol”. Subsequently, the enrichment results were visualized using a microbiology platform, and the top 20 most significant GO enrichment terms and KEGG pathways were presented in the form of bubble plots (*P* < 0.05).

### 2.7 Identification of targets and pathways for HUA treatment by SLJZ

The intersection of DEGs and targets obtained from network pharmacology was used to identify the action targets of SLJZ for HUA. Additionally, the results of GO and KEGG analyses were integrated to elucidate the potential pathways of SLJZ in treating HUA.

### 2.8 Molecular docking

The Autodock Vina was used for molecular docking to explore possible interaction models between active compounds and core target proteins.The crystal structure of the core target protein was retrieved from the RCSB-PDB database (https://www.rcsb.org). Preprocessing of the protein receptor involved the removal of ligands, water and the addition of hydrogen. The 3D structure of the active compound ligand was obtained from the PubChem database. Subsequently, molecular docking of the modified binding site with the ligand was conducted using Autodock Vina, and the resulting docking poses were visualized using PyMOL software.

### 2.9 Determination of DA, IL-6, IL-1β and TNF-α in the kidneys

PBS solution containing protease inhibitors was added to the mouse kidney tissue, followed by homogenization. The mixture was then centrifuged at 5000 rpm for 10 min, and the supernatant was collected. The concentrations of DA, IL-6, IL-1β, and TNF-α in the supernatant were measured using specific kits according to the manufacturer’s instructions.

### 2.10 Immunohistochemistry staining

Kidney tissue sections were analyzed using immunohistochemistry (9). The sections were placed in a citric acid-based repair solution (pH=6.0), within an autoclave, heated until bubbling, and maintained for 2 min. After natural cooling, the sections were incubated in 3% H_2_O_2_ at room temperature for 20 min to block endogenous peroxidase activity. Subsequently, the sections were sequentially incubated with primary and secondary antibodies for further analysis. Positive staining on each image was quantified by Image J software.

### 2.11 Statistical analysis

Data analysis was conducted using GraphPad Prism 8.0 software. Results are presented as mean ± standard deviation (SD). Intergroup comparisons were performed using one-way ANOVA, and *P* < 0.05 were considered to indicate statistical significance.

## 3 Results

### 3.1 SLJZ reduced UA levels in HUA mice

**Figure 1:**
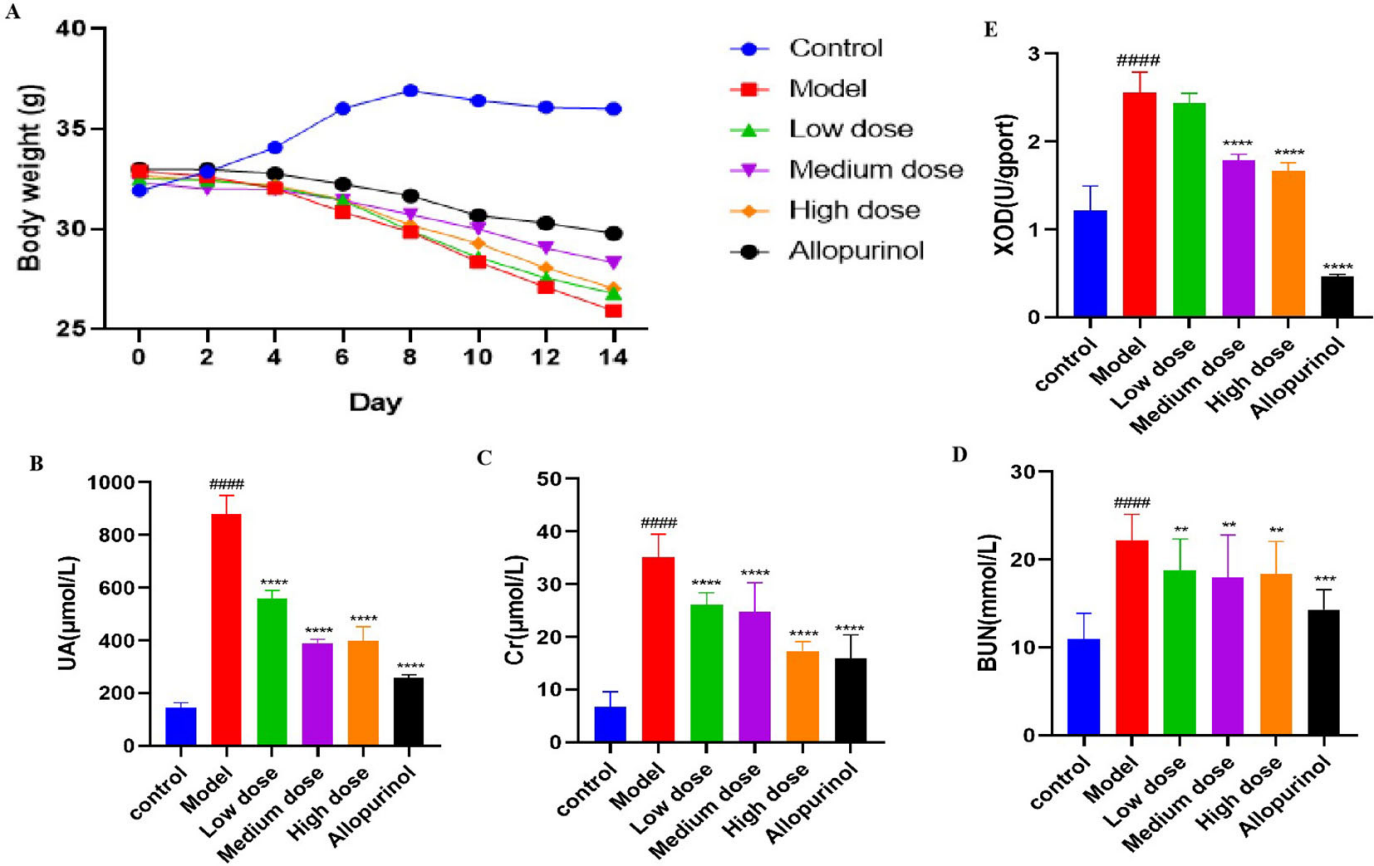
Effects of SLJZ treatment on HUA mice. **(A)** Changes in body weight of mice. **(B)** UA level. **(C)** Cr level. **(D)** BUN level. **(E)** XOD level. Note: Data were expressed as mean ± SD (n = 8). # indicates comparison with the control group, ^####^ *P*<0.0001, * indicates comparison with the model group, ^**^ *P*<0.01, ^***^ *P*<0.001, ^****^ *P*<0.0001.

From the completion of adaptive feeding to the end of drug administration on day 14, the body weights of the mice were continuously recorded (**Figure 1A**). We observed that the body weights of mice in the control group increased significantly and stabilized at a certain level, whereas the mice that had been modeled all began to decrease significantly starting from the fourth day. the model group exhibited the most pronounced weight loss, while the AP group showed the least reduction. The significant reduction in body weight observed in both the low-dose and high-dose SLJZ groups may be explained by the nature of SLJZ as a drug for hyperlipidemia treatment. At the low dose, its insufficient renal protective effect might contribute to this outcome, whereas at the high dose, its pronounced lipid-lowering efficacy likely dominates the observed weight changes. In addition to body weight reduction, the modeled mice exhibited decreased appetite, disheveled fur, depression, and in severe cases, prolonged reflexes. Following drug intervention, all of these conditions were significantly improved.

To evaluate the therapeutic effect of SLJZ on HUA, blood samples from mice in each group were collected at the end of modeling on day 14, and serum centrifugation was performed the following day to detect HUA-related indices. Compared with the control group, the UA levels in the model group were significantly elevated, confirming the successful establishment of the HUA model (10). The UA levels in treated mice decreased significantly (**Figure 1B**). Renal function was assessed using Cr and BUN as markers. We found that Cr levels in the model group were significantly higher than those in the control group, whereas Cr levels in the high-dose SLJZ group were reduced to levels comparable to those in the AP group and significantly lower than those in the model group (**Figure 1C**). Although BUN levels were similar across the low, medium, and high-dose SLJZ groups, they were all significantly lower than those in the model group (**Figure 1D**). XOD is a key enzyme in UA production, converting hypoxanthine to xanthine and subsequently producing UA (11). **Figure 1E** shows the XOD levels in each group of mice. Compared with the control group, XOD levels in the Mode group were significantly increased. While there was no significant difference between the low-dose SLJZ group and the model group, XOD levels in the medium and high-dose SLJZ groups, as well as the AP group, were significantly lower. Notably, XOD levels in the AP group were even lower than those in the control group. It may be because AP competitively binds to XOD, thereby reducing UA production; however, the strong inhibitory effect of AP on XOD may lead to the accumulation of hypoxanthine and xanthine in the body, potentially causing kidney injury. The results indicate that SLJZ effectively inhibits UA accumulation in HUA mice and provides renal protection.

### 3.2 Kidney pathological evaluation

**Figure 2:**
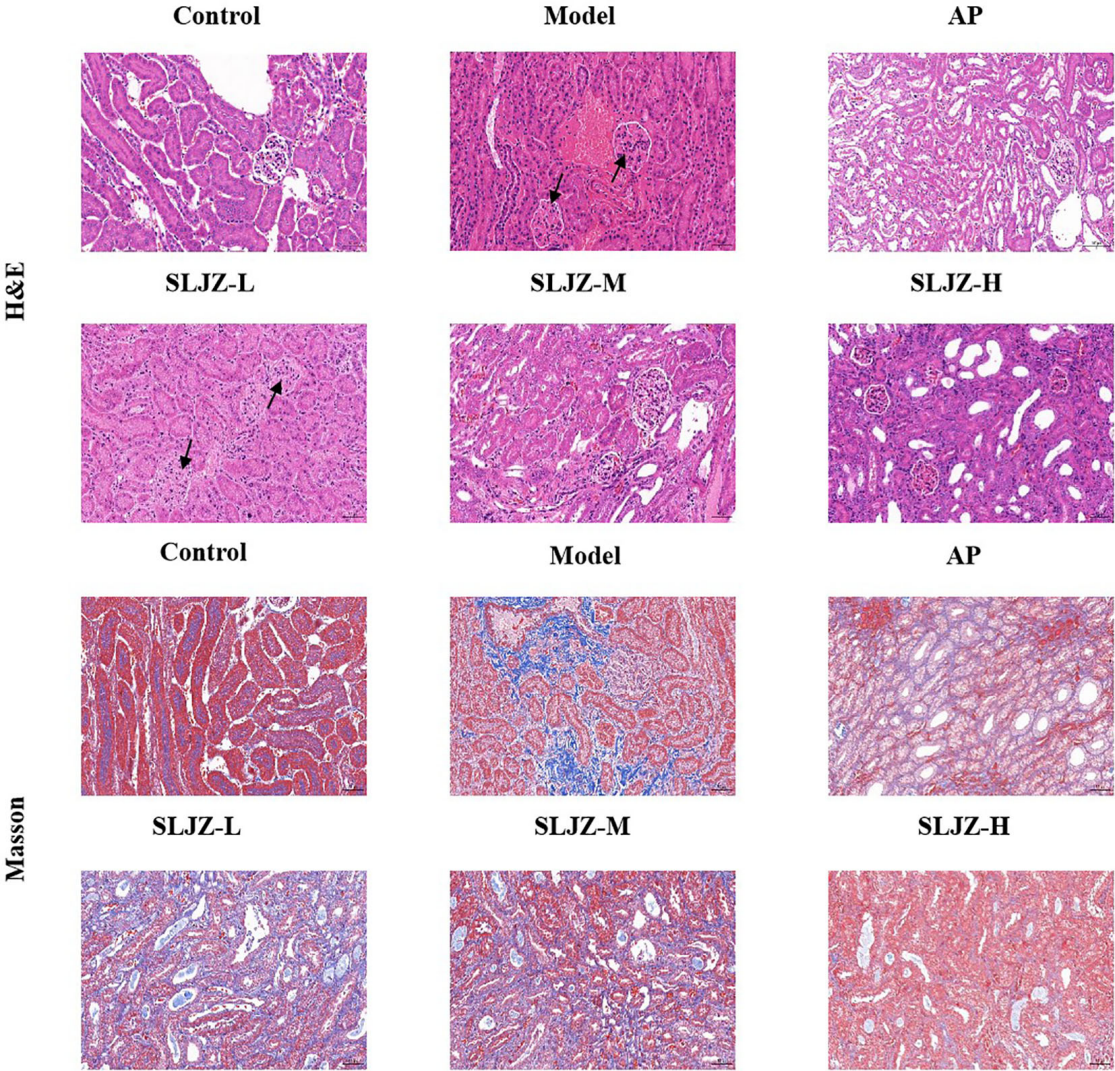
Effect of SLJZ on renal histopathology in HUA mice, H&E staining and Masson staining showed the protective effect of SLJZ on the kidney. Magnification 200×, scale bar 50 μm. black arrows indicate glomerular lesions.

The progression of HUA is frequently associated with renal damage; therefore, histologic changes in the kidneys were evaluated using H&E staining (**Figure 2**). The kidneys of mice in the control group exhibited normal structural integrity, with clearly defined glomeruli and well-organized renal tubular structures. In contrast, the renal tissues of mice in the model group displayed pronounced pathological alterations, including degeneration and necrosis of renal tubular epithelial cells, as well as increased inflammatory cell infiltration in the renal interstitium. These pathological changes were significantly ameliorated following interventions with SLJZ and AP.

We also evaluated the role of SLJZ in HUA-induced renal tissue fibrosis using Masson staining (**Figure 2**). There were fewer blue collagen fibers in the renal tubular interstitium of mice in the control group, and a few collagen fibers were visible in the perivascular area of the blood vessels in the model group whereas there was a significant increase in blue collagen fibers in the renal interstitium. In contrast, both SLJZ and AP treatments significantly reduced collagen deposition in renal tubular interstitium. These findings indicate that SLJZ effectively alleviates renal pathologic changes associated with HUA-induced fibrosis.

### 3.3 Network pharmacology-based analysis

#### 3.3.1 Target prediction of blood entry components

**Figure 3:**
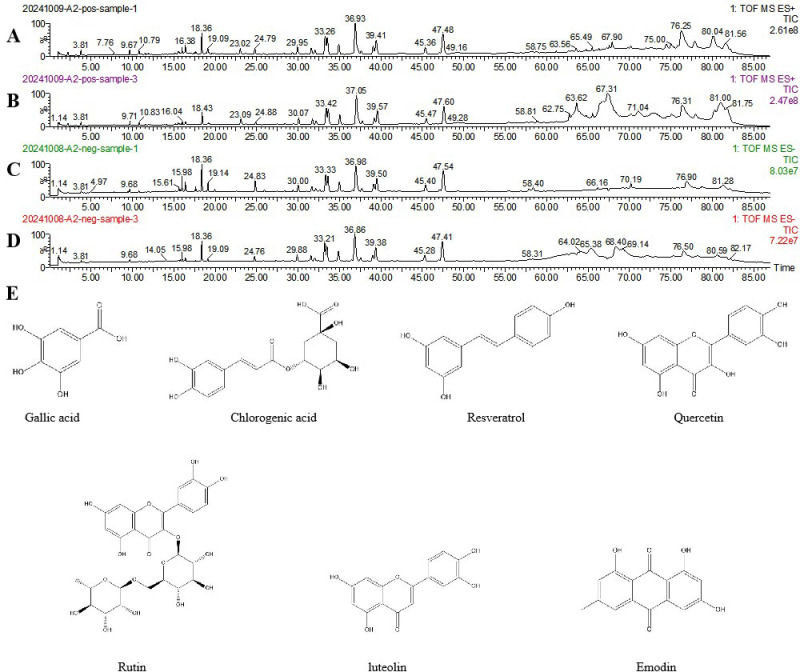
Total ion flow plots of SLJZ blood-entry components in positive and negative ion modes as determined by UPLC-Q-TOF-MS. **(A)** Model group serum in positive ion mode. **(B)** SLJZ-containing serum in positive ion mode. **(C)** Model group serum in negative ion mode. **(D)** SLJZ-containing serum in negative ion mode. **(E)** Structures of compounds identified in SLJZ.

**Figure 4:**
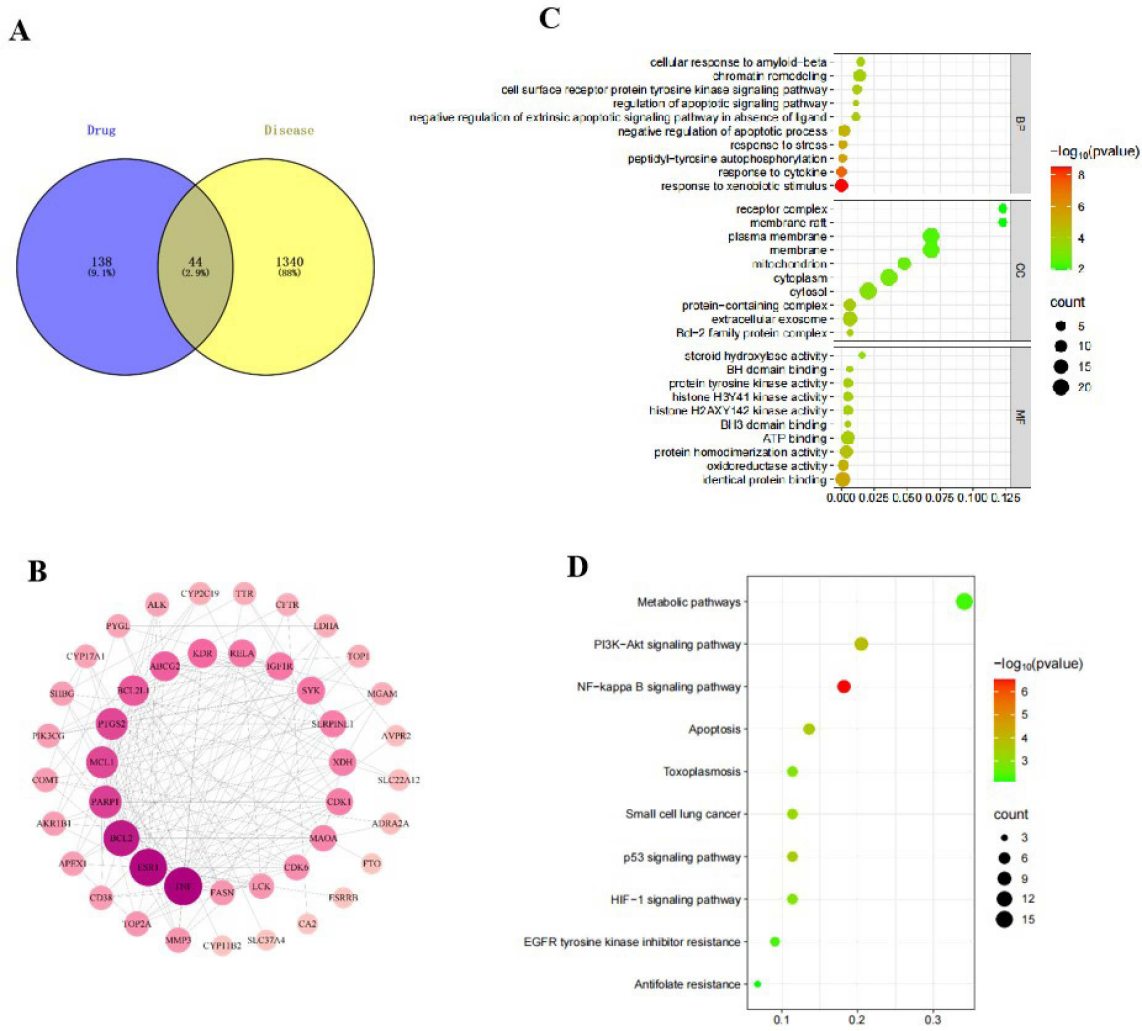
**(A)** Wayne diagram of the intersection of drug targets and disease targets. **(B)** PPI network of intersecting targets. **(C)** GO function enrichment analysis bubble diagram. **(D)** KEGG pathway enrichment analysis bubble diagram.

We initially investigated the potential mechanisms of action and targets involved in the treatment of HUA with SLJZ based on the blood-entry components. By comparing the primary mass spectra of blank serum and SLJZ-containing serum (**Figure 3**), a total of 14 blood-entry components were identified, and 7 of these were selected for further analysis (**Table 1**). Through extensive database searches, we identified 182 drug-related targets and 1,384 disease-related targets after removing duplicates. Venn diagrams revealed 44 overlapping targets between drug and disease targets, indicating that these targets may serve as potential therapeutic targets for SLJZ in the treatment of HUA (**Figure 4A**).

**Table 1:** Blood Ingredients.

No	Component identification	MF	Rt (min)	Addition ion	Incoming blood fragment ion (*m/z*)	Literature sources
1	Gallic acid	C_7_H_6_O_5_	1.984	[M+1]^+^	127.0501, 109.1019	Liu et al., (12)
2	Chlorogenic acid	C_16_H_18_O_9_	10.174	[M-1]^-^	191.0160, 161.1194	Tang et al., (13)
3	Resveratrol	C_14_H_12_O_3_	17.346	[M+1]^+^	228.1980, 211.0953, 136.1124, 108.0804	Xu et al., (14)
4	Rutin	C_27_H_30_O_16_	28.829	[M+1]^+^	453.1614, 255.1566	Wu et al., (15)
5	Quercetin	C_15_H_10_O_7_	41.207	[M-1]^-^	151.2979, 124.1860, 107.4699	Meng et al., (16)
6	luteolin	C_15_H_10_O_6_	46.139	[M-1]^-^	285.0325, 267.0573, 257.2115, 241.0722	Yu et al., (17)
7	Emodin	C_15_H_10_O_5_	56.608	[M+1]^+^	255.0618, 241,1761, 213.1430, 185.1137	Hou et al., (18)

To investigate the interactions among these 44 overlapping targets, the PPI network of potential targets was constructed and analyzed using Ctoscape 3.9.1 software. In the network, nodes with higher degree counts were highlighted with darker red color and larger dot size (**Figure 4B**). As shown in the figure, the genes with higher degree values include TNF, ESR1, BCL2, PARP1 and MCL1. Among these, TNF is an inflammatory factor, ESR1, BCL2 and MCL1 are closely associated with apoptosis regulation, and PARP1 is a key protein involved in DNA damage response. Notably, DNA damage can induce oxidative stress in cells, which in turn activates the expression of death receptors, thereby initiating the apoptotic process. These findings suggest that the therapeutic effects of SLJZ on HUA may involve anti-inflammatory, antioxidant, and anti-apoptotic mechanisms.

#### 3.3.2 GO and KEGG analysis

To investigate the pathways of action of SLJZ in treating HUA, 44 potential targets were subjected to GO and KEGG enrichment analysis. The top 10 significantly enriched terms from GO enrichment analysis in the categories of BP, CC, and MF are presented in **Figure 4C**. The results revealed that BP was primarily associated with response to xenobiotic stimulus and negative regulation of apoptotic process, etc.; CC was predominantly distributed in extracellular exosome and protein-containing complex, etc.; MF is mainly involved in identical protein binding and oxidoreductase activity, etc. Additionally, the top 10 enriched signaling pathways, including the NF-kappa B signaling pathway, PI3K-Akt signaling pathway, and apoptosis-related pathways, are illustrated in **Figure 4D**, suggesting that SLJZ’s treatment of HUA may involve anti-inflammatory and oxidative stress-related mechanisms.

### 3.4 RNA-seq and data analysis

#### 3.4.1 DEGs screening

**Figure 5:**
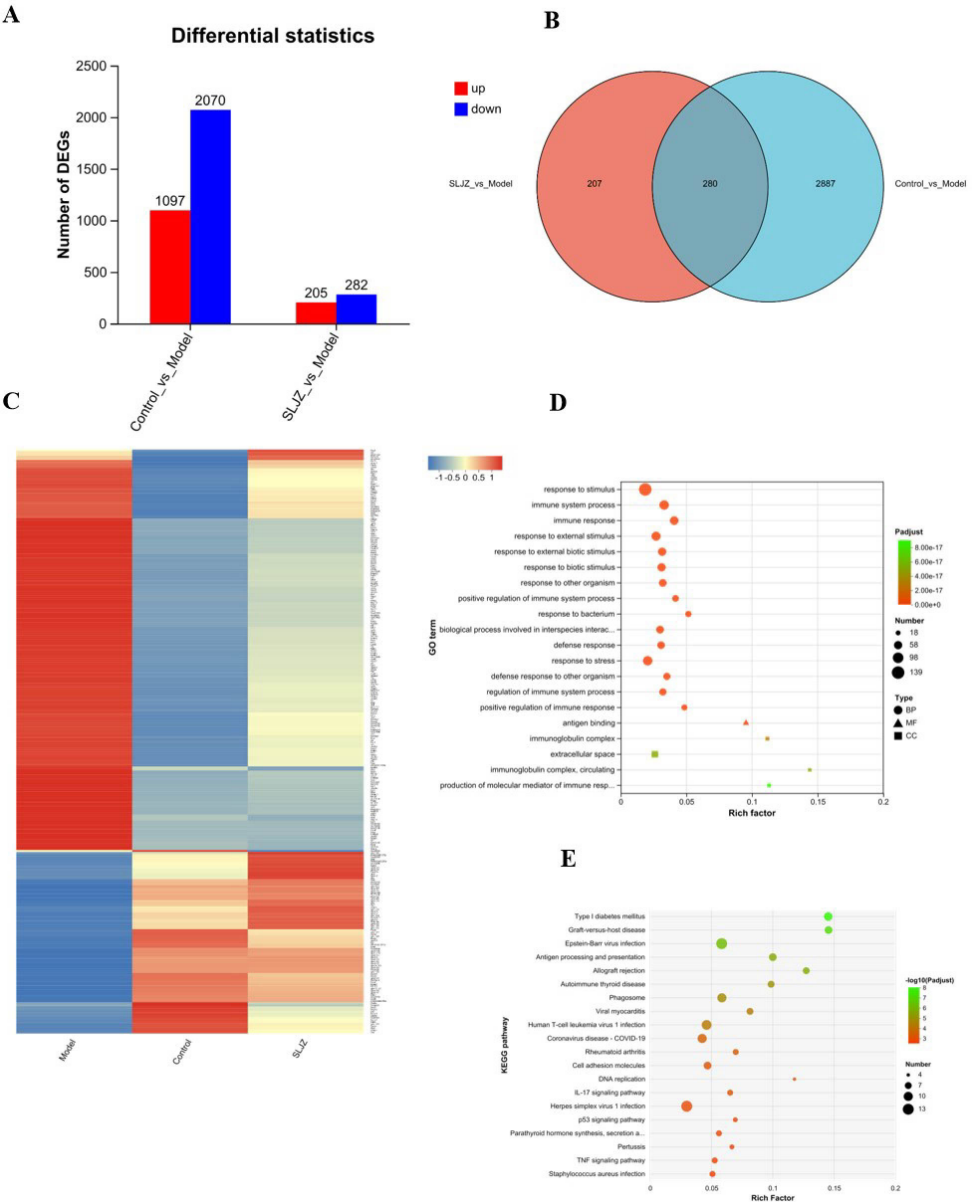
Differential gene expression patterns. **(A)** Statistics on the number of the Up/Down-regulated genes in control vs model and SLJZ vs Model groups. **(B)** Venn diagram showed the number of the intersection genes between the model vs control and SLJZ vs model groups. **(C)** Heatmap of hierarchical clustering showed the 280 HUA-related DEGs of SLJZ in the control, model, and SLJZ groups. Color intensity was proportional to the abundance of gene expression. Red represented up-regulated genes and blue represented down-regulated genes. **(D)** GO enrichment of 280 DEGs with legend shapes distinguishing the three functional classes BP, CC and MF. **(E)** Bubble diagram of the top ten KEGG-significant pathways of DEGs (*P*<0.05).

We employed transcriptome sequencing to further investigate the mechanisms of action and targets of SLJZ in treating HUA. As shown in **Figure 5A**, a total of 3167 DEGs were identified with |log2 FC| ≥ 1 and *P* ≤ 0.05, including 1097 up-regulated and 2070 down-regulated genes between the model and control groups. Similarly, 487 DEGs were obtained between the SLJZ and model groups, with 205 genes up-regulated and 282 genes down-regulated. In **Figure 5B**, the overlap of DEGs between the model vs. control and SLJZ vs. model groups indicates that 280 DEGs were either up- or down-regulated by HUA but inversely regulated by SLJZ. In **Figure 5C**, neural network clustering of these 280 DEGs was performed across the control, model, and SLJZ groups, clearly demonstrating the reversal of DEGs in the kidney in response to SLJZ treatment.

#### 3.4.2 GO and KEGG analysis

The DEGs were subjected to GO enrichment analysis, and the results are presented in **Figure 5D**, with circles representing BP, triangles representing MF, and squares representing CC. As shown in the figure, among the top 20 entries, only 1 entry was associated with MF (antigen binding), and 3 entries were related to CC, including immunoglobulin complex, extracellular space, and immunoglobulin complex, circulating, suggesting that SLJZ ‘s uric acid-lowering efficacy may be linked to cellular immune responses. Additionally, there were 16 entries for BP, which included response to stimulus, immune system process, immune response, etc. Taken together, these findings indicate that the immune response was significantly activated during the HUA process. The inflammatory response, as a results of heightened immunity, inter-regulated with immune function to maintain the homeostasis in the human body (19). This suggests that SLJZ may exert therapeutic effects on HUA by inhibiting the expression of inflammatory factors (20), which is consistent with previous web pharmacology results.

Further KEGG pathway analysis revealed that the 280 DEGs were annotated to 188 pathways, among which 50 pathways were significantly enriched (*P* < 0.05). **Figure 5E** displays the top 20 most significant pathways, which include 12 disease-related pathways and various biological information processing processes. By integrating relevant literature, we identified that the pathways related to HUA include the IL-17 signaling pathway (21), the p53 signaling pathway (22), and the TNF signaling pathway (23). These pathways are primarily involved in inflammatory responses and oxidative stress regulation, suggesting that anti-inflammation and antioxidation mechanisms may be critical for SLJZ to exert its uric acid-lowering activity.

### 3.5 Mining of core targets and pathways of action

Previously, we predicted the potential targets of SLJZ for treating HUA using network pharmacology. Subsequently, transcriptomics was employed to identify the differential genes reversed by SLJZ in the kidney tissues of HUA mice, thereby preliminarily elucidating the mechanisms underlying SLJZ’s uric acid-lowering effects, which involve anti-inflammatory and antioxidant activities. To further explore the core targets of SLJZ in HUA treatment, we overlapped the 44 potential targets predicted by network pharmacology with the 280 DEGs identified through transcriptomics. This analysis revealed two intersecting targets: COMT and SERPINE1. These are key targets of SLJZ in HUA treatment, and their expression levels are presented in **Table 2** (compared with the model group).

**Table 2:** Expression levels of core proteins.

Gene ID	Gene name	|log2 FC|	p*-*value	Significant	Regulate
ENSMUSG00000000326	COMT	1.2457161391399998	0.00185875409532	yes	down
ENSMUSG00000037411	SERPINE1	1.8349896000900001	0.0045249185033	yes	down

COMT is primarily involved in dopamine metabolism process *in vivo*, acting synergistically with MAOA (24). Notably, MAOA has already been identified in previous web-based pharmacological predictions. Dopamine can promote glomerular filtration *in vivo*, thereby enhancing urate excretion and contributing to UA reduction. Conversely, the up-regulation of COMT and MAOA decreases dopamine levels *in vivo*, inhibiting renal tubular filtration and impeding UA salt excretion. SERPINE1 serves as the primary inhibitor of urokinase. In conjunction with prior experimental results, urate accumulation *in vivo* activates NLRP3 inflammasomes, while SERPINE1, as a regulator of inflammatory responses, exacerbates inflammation by suppressing the fibrinolytic system. Additionally, within the p53 signaling pathway, the upregulation of SERPINE1, a downstream protein of p53, further intensifies oxidative damage (25), which subsequently elevates UA levels *in vivo*. Based on these findings, we selected COMT, MAOA, p53, NLRP3, and SERPINE1 as the core targets for validating SLJZ’s efficacy in treating HUA.

### 3.6 The molecular docking results of COMT, MAOA, p53, NLRP3 and SERPINE1 with active compounds

**Figure 6:**
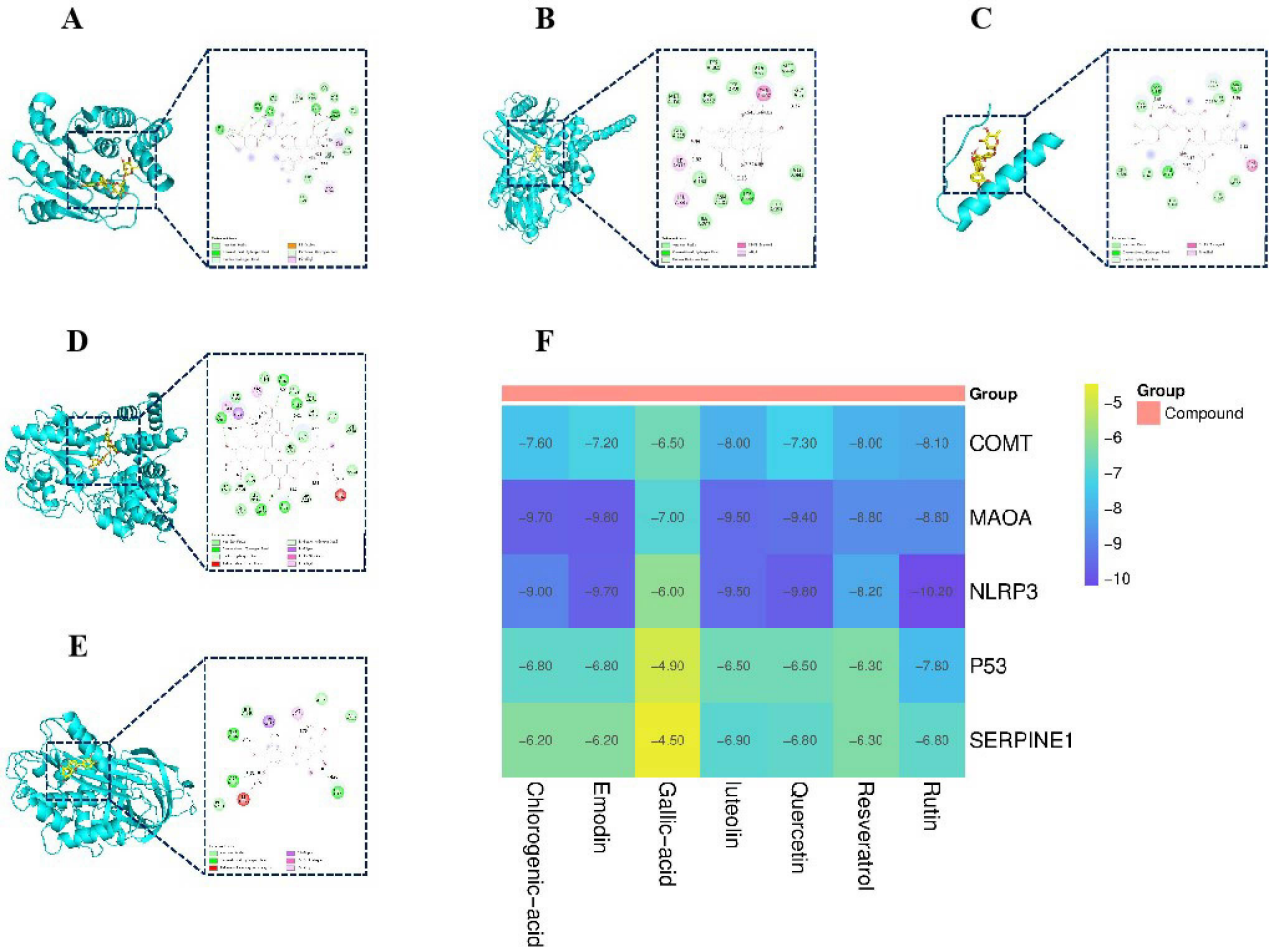
Molecular docking results. **(A)** Binding mode of COMT- rutin complex. **(B)** Binding mode of MAOA- emodin complex. **(C)** Binding mode of p53- rutin complex. **(D)** Binding mode of NLRP3- rutin complex. **(E)** Binding mode of SERPINE1- luteolin complex. **(F)** Binding energy heat map.

To verify the interaction between the active compounds and core targets, molecular docking was performed to evaluate the binding energy between small molecules and target proteins. **Figures 6A–E** display the compounds with the highest docking scores for COMT, MAOA, p53, NLRP3, and SERPINE1, respectively. The binding energy heatmap is presented in **Figure 6F**. The results demonstrate that the seven active compounds exhibit favorable binding affinities with COMT, MAOA, p53, NLRP3, and SERPINE1.

### 3.7 The levels of DA, IL-6, IL-1β and TNF-α in the kidneys of mice

**Figure 7:**
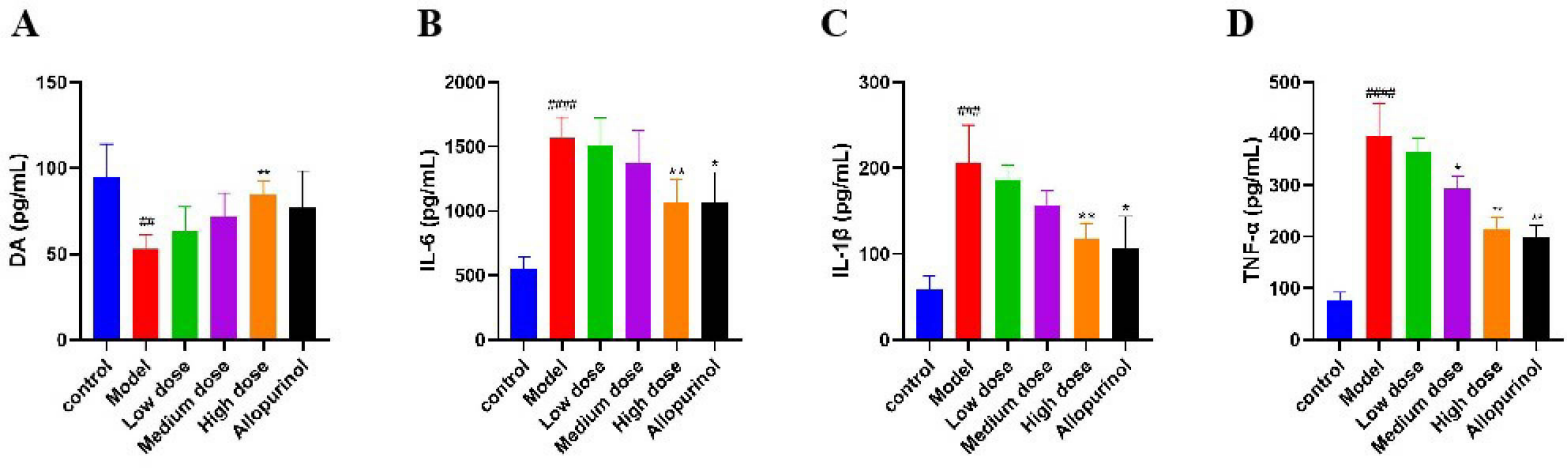
Regulation of SLJZ on DA and inflammatory factors. **(A)** DA level. **(B)** IL-6 level. **(C)** IL-1β level. **(D)** TNF-α level. Data were expressed as mean ± SD (n = 6). # indicates comparison with the control group, ^##^ *P*<0.01, ^###^ *P*<0.001, ^####^ *P*<0.0001, * indicates comparison with the model group, ^*^ *P*<0.05, ^**^ *P*<0.01.

COMT and MAOA are the critical rate-limiting enzymes in the DA metabolic process. Their upregulation promotes the degradation of DA, thereby inhibiting UA excretion. Upon activation, NLRP3 induces the release of IL-6, IL-1β, and TNF-α. Therefore, measuring the levels of DA, IL-6, IL-1β, and TNF-α in the kidneys of mice can indirectly reflect the regulation of COMT, MAOA, p53, NLRP3, and SERPINE1 by SLJZ. Compared with the control group, the DA content in the kidneys of the model group was significantly reduced, indicating that the DA metabolic process was accelerated. This effect was ameliorated after SLJZ intervention (**Figure 7A**). Compared with the control group, the levels of IL-6, IL-1β, and TNF-α in the kidneys of the model group were significantly elevated. In the high-dose group, the levels of IL-6, IL-1β, and TNF-α were decreased to levels comparable to those of the AP group and were significantly lower than those of the model group (**Figures 7B–D**).

### 3.8 Immunohistochemical study of the effect of SLJZ on protein expression of COMT, MAOA, p53, NLRP3 and SERPINE1 in HUA mice

**Figure 8:**
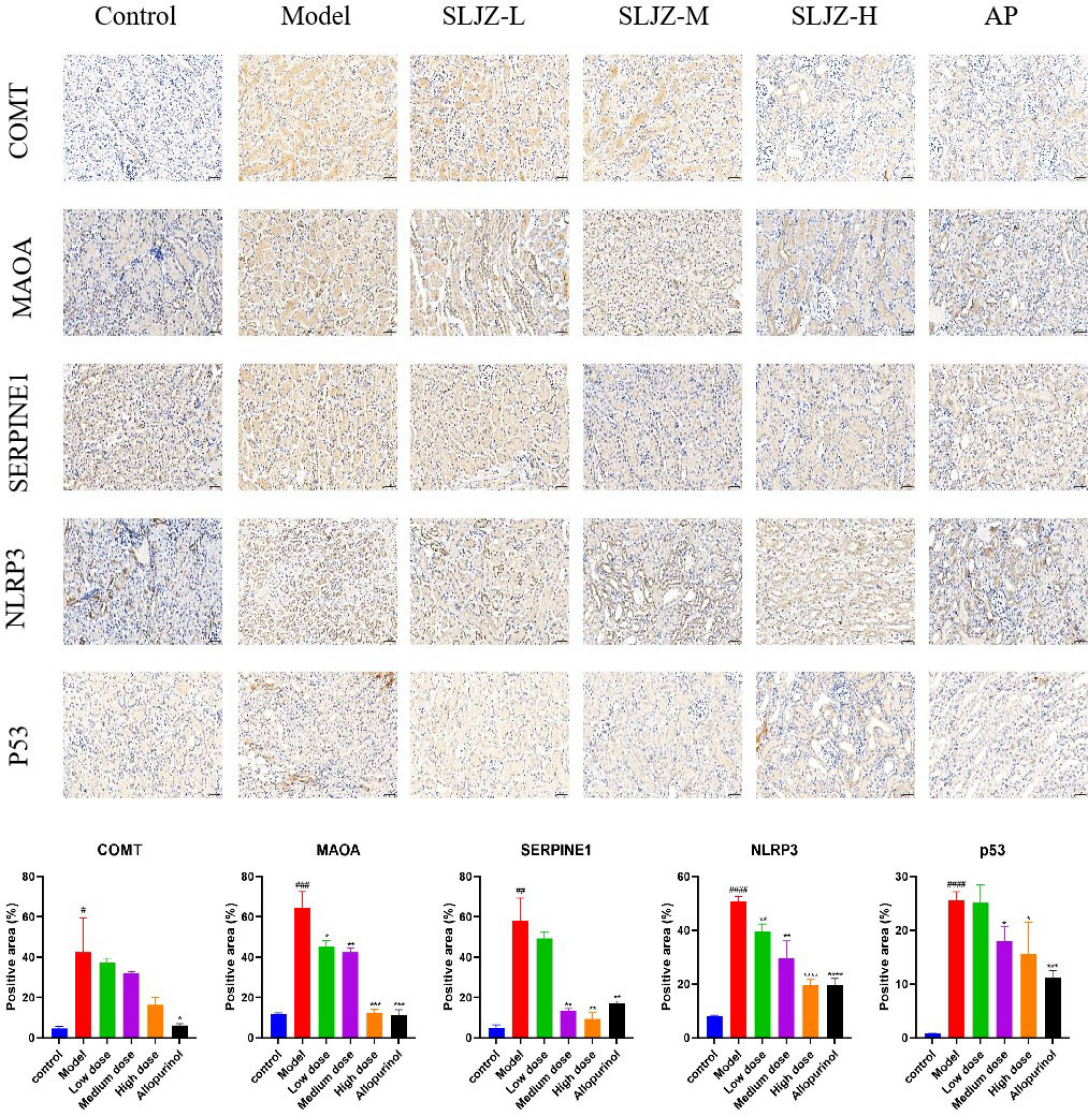
Histological images of immunohistochemical staining for COMT, MAOA, SERPINE1, NLRP3 and p53 and quantification of positively stained areas. Magnification 200×, scale bar 50 μm. # indicates comparison with the control group, ^#^ *P*<0.05, ^##^ *P*<0.01, ^###^ *P*<0.001, ^####^ *P*<0.0001, * indicates comparison with the model group, ^*^ *P*<0.05, ^**^ *P*<0.01, ^***^ *P*<0.001, ^****^ *P*<0.0001.

The results of the previous experiments suggested that SLJZ might exert its uric acid-lowering effect by regulating the COMT/MAOA signaling pathway and the p53/SERPINE1/NLRP3 signaling pathway. Therefore, the expression levels of the relevant proteins in renal tissues were examined using immunohistochemistry, and the results are presented in **Figure 8**. In the immunohistochemical analysis, the target proteins appeared as yellowish-brown staining. As can be clearly observed from the figure, the expression levels of COMT, MAOA, SERPINE1, NLRP3, and p53 in the model group were significantly higher than those in the control group, and these proteins were down-regulated after SLJZ intervention. These findings indicated that SLJZ may reduce UA levels by inhibiting the COMT/MAOA signaling pathway and the p53/SERPINE1/NLRP3 signaling pathway.

## 4 Discussion

The kidney, as a critical organ for UA excretion, plays a pivotal role in maintaining UA homeostasis in the body. Approximately 2/3 of the body’s UA is excreted via the kidneys. Elevated UA levels, often attributed to reduced renal excretion of UA, can lead to HUA (26). Patients with HUA frequently require long-term pharmacological intervention, and in some cases, lifelong management. However, commonly prescribed drugs such as allopurinol, febuxostat, and benzbromarone are associated with significant adverse effects, limiting their efficacy in treating HUA (27). SLJZ, known for its lipid-lowering, hepatoprotective, nephroprotective properties, and minimal side effects, has been widely applied in the treatment of endocrine and metabolic disorders for many years. In this study, we used transcriptomics and network pharmacology to comprehensively investigate the potential targets and mechanisms of SLJZ in managing HUA from a holistic perspective, thereby providing a robust theoretical foundation for its clinical application.

According to the pathogenesis of HUA, UA was directly administered to increase UA influx, while potassium oxalate was used to inhibit UA excretion, thereby establishing a stable HUA mouse model. After 14 days of treatment, the levels of UA, Cr, BUN were reduced to varying degrees in both the SLJZ and AP groups. Moreover, SLJZ demonstrated a moderate inhibitory effect on XOD, which may help mitigate the adverse effects associated with excessive XOD inhibition by AP. Histopathological analysis revealed that SLJZ could alleviate renal interstitial inflammatory cell infiltration and inhibit the fibrotic process in renal tissue, thereby reducing renal injury. In conclusion, SLJZ exhibits promising therapeutic effects for HUA and effectively alleviates renal injury induced by HUA.

After being absorbed into the bloodstream, the active ingredients of Chinese medicines must reach a certain blood concentration to exert their pharmacological effects. Therefore, the components entering the bloodstream are considered the key active ingredients responsible for pharmacological actions (28). Network pharmacology can systematically explore the connections between drugs and diseases from a holistic perspective, elucidating the multi-component and multi-target mechanisms of traditional Chinese medicine (29). In this study, we used UPLC-Q-TOF-MS to identify the blood-entry components of SLJZ and combined with network pharmacology methods to predict its potential targets and related pathways in treating HUA. Seven components—chlorogenic acid, gallic acid, quercetin, luteolin, rutin, resveratrol and emodin—were identified as the active components of SLJZ for HUA treatment. Through network pharmacological analyses, 44 potential targets were identified, and the therapeutic effects of SLJZ on HUA were preliminarily attributed to its anti-inflammatory, antioxidant, and anti-apoptotic properties. RNA-seq results revealed 280 DEGs that were inversely regulated by SLJZ intervention in HUA. Functional enrichment analysis showed that GO terms were primarily enriched in immune response-related biological processes. In the context of HUA, urate crystals accumulate in renal tissues and may be perceived as foreign bodies, triggering a robust immune response and inflammation. KEGG pathway enrichment results further confirmed involvement in inflammatory responses and oxidative stress, suggesting that SLJZ’s mechanism of action in treating HUA may involve anti-inflammatory and antioxidant pathways, which aligns with the findings from network pharmacology.

To clarify the key targets and mechanisms of action of SLJZ for HUA, two targets, COMT and SERPINE1, were identified by intersecting network pharmacology and RNA-seq results. COMT is an enzyme that catalyzes the methylation of catecholamine neurotransmitters and plays a synergistic role in DA metabolism, primarily in conjunction with MAOA (30). DA, an essential neurotransmitter in the central nervous system, activates renal tubular D1 receptors, dilates renal vasculature, and increases renal blood flow, thereby promoting urate excretion (31). Moreover, DA indirectly modulates inflammatory response by regulating immune cell function, such as inhibiting T-cell activation (32), which aligns with the findings from GO enrichment analysis. Certain animal experiments have demonstrated that dysfunctions in the dopaminergic system exacerbate HUA (33). Consequently, although COMT and MAOA are not directly involved in UA metabolism, they can indirectly regulate UA levels *in vivo* through their effects on energy metabolism and inflammatory responses.

SERPINE1, as a fibrinogen activator inhibitor, is also considered a major inhibitor of urokinase. By inhibiting urokinase activity, SERPINE1 reduces glomerular filtration and it thereby disrupts UA homeostasis *in vivo*. HUA, characterized as a low-grade inflammatory state, leads to the accumulation of UA crystals in the kidneys. This triggers the activation of NLRP3 inflammasomes, promoting the release of pro-inflammatory cytokines such as IL-6, IL-1β, and TNF-α (34). SERPINE1, acting as a regulator of the inflammatory response, is significantly upregulated in inflammatory environment. By inhibiting the fibrinolytic system (e.g., fibrinogenesis), SERPINE1 exacerbates the inflammatory response, creating a vicious cycle. The p53 signaling pathway, identified as the highest-scoring SERPINE1-related pathway in both network pharmacology and transcriptomics analyses, plays a critical role in HUA-induced oxidative stress. Oxidative stress activates the p53 signaling pathway, leading to apoptosis. As an upstream regulator of SERPINE1, p53’s upregulated expression further amplifies oxidative damage, forming a positive feedback loop. Studies have shown that in renal cells treated with UA, the co-expression of p53 and SERPINE1 is significantly increased. This can be partially reversed by antioxidants (35).

The molecular docking results demonstrated that the seven active components exhibited strong binding affinities to COMT, MAOA, SERPINE1, NLRP3, and p53. Notably, rutin displayed the lowest binding energy with NLRP3 at -10.2 kcal/mol, indicating that SLJZ could regulate these proteins through direct interactions. Additionally, SLJZ was found to increase DA levels in the kidneys of mice while decreasing the levels of IL-6, IL-1β, and TNF-α, further supporting this conclusion. Based on these findings, we identified COMT, MAOA, SERPINE1, NLRP3, and p53 as the core targets for SLJZ’s therapeutic effects on HUA and validated them using immunohistochemistry. The results revealed that SLJZ exerted anti-inflammatory and antioxidant effects by downregulating the expression of COMT, MAOA, SERPINE1, NLRP3, and p53, thereby achieving its therapeutic effect on HUA.

## 5 Conclusion

In conclusion, the present study demonstrated through pharmacological experiments that SLJZ could effectively reduce the levels of UA, Cr, and BUN, inhibit XOD activity, and alleviate renal damage in HUA mice. By integrating network pharmacology, transcriptomics, and molecular docking, we revealed that SLJZ exerts its therapeutic effects on HUA by inhibiting the COMT/MAOA signaling pathway and the p53/SERPINE1/NLRP3 signaling pathway. The relevance of DA metabolism, inflammatory inhibition and oxidative stress to HUA was further highlighted. This comprehensive research approach not only provides theoretical support for the clinical application of SLJZ in treating HUA but also lays a solid foundation for further exploration of its therapeutic potential.

## Data Availability

The datasets for this manuscript are not publicly available because these are required for ongoing studies. Requests to access the datasets should be directed to corresponding author.
